# XPF–ERCC1: Linchpin of DNA crosslink repair

**DOI:** 10.1371/journal.pgen.1008616

**Published:** 2020-04-09

**Authors:** Peter J. McHugh

**Affiliations:** Department of Oncology, MRC Weatherall Institute of Molecular Medicine, University of Oxford, John Radcliffe Hospital, Oxford, United Kingdom; University of Washington School of Medicine, UNITED STATES

A spectacularly toxic form of damage, the interstrand crosslink (ICL), arises when two strands of duplex DNA become covalently linked [1]. The cellular response to ICLs is of great interest because several antitumour drugs, including platinum agents and mitomycin C, kill cancer cells by inducing ICLs [2, 3]. More recently it has become apparent that metabolites continually burden cells with ICLs, which must be removed for cells to maintain function and viability. The importance of efficient ICL repair in development and health is illustrated by the clinical features of a devastating inherited syndrome, Fanconi anemia (FA), which is thought to be the result of defective ICL repair [4]. FA patients suffer from bone marrow failure, leukaemia, and solid malignancies. While the endogenous DNA-crosslinking agent, or agents, responsible for the damage that lies unrepaired in the cells of FA patients are unknown, suspects include the common metabolites formaldehyde and acetaldehyde (including that derived from ingested alcohol) and oxidised lipid species [5–7].

The machinery available to repair ICLs has expanded markedly through evolution. Both *Escherichia coli* and yeasts almost exclusively rely on a modified form of nucleotide excision repair (NER) [1, 8], a cut-and-paste pathway that is best known for its ability to remove UV-light–induced DNA photodimers (whose defects result in another human syndrome, Xeroderma pigmentosum [XP]). The details of how modified NER removes ICLs remain obscure and merit further investigation. Metazoans, meanwhile, have developed additional pathways for ICL repair—most significantly, an ‘FA pathway’ [4]. The functional characterization of genes and factors defective in FA patients (22 genes to date) have revealed a repair pathway that operates during DNA replication and possibly elsewhere in the cell cycle (although this aspect has yet to be explored systematically). Although how the FA pathway coordinates ICL repair is still subject to intense study, it appears to orchestrate initial responses to ICLs, some steps of the nucleolytic processing of ICLs, and the subsequent resolution of the incised intermediates through the sequential action of translesion-synthesis polymerases and homologous recombination reactions [9]. For mammalian cells, it is not clear whether there is a genetic or functional relationship between the NER and FA pathways in response to ICLs. Here, Mulderrig and Garaycoechea show that while both the FA and NER pathways both play important roles in response to ICLs, additional pathways also contribute to their repair [10].

At the heart of this uncertainty about the contributions of the FA and NER pathways to ICL repair is a dimeric endonuclease, XPF–ERCC1, required for both pathways and mutated in both FA (complementation group Q, FANCQ) and XP (complementation group F) [11, 12]. XPF–ERCC1 has received much attention, since it was realised several decades ago that XPF–ERCC1-deficient mammalian cells are exquisitely sensitive to ICL-inducing agents [13, 14], showing a sensitivity that exceeds that observed in other NER-deficient and FA-deficient cells. Moreover, there is a consensus from cellular and biochemical studies that XPF–ERCC1 is the major activity responsible for making the endonucleolytic DNA incisions that initiate ICL repair [15–17]. Mulderrig sought to deduce whether the extreme ICL sensitivity of XPF–ERCC1 defective cells is the result of the combined loss of the FA and NER pathways or whether XPF–ERCC1 makes contributions to additional repair pathways.

Defining the division of labour between the NER and FA pathways is complicated by several factors. First, the NER pathway can be divided into two subpathways: the transcription-coupled NER component (TC-NER) required for the efficient repair of active genes, triggered by RNA polymerase II arrest; and global genome NER (GG-NER) that operates throughout the genome [18, 19]. Fortunately, these subpathways can be distinguished by the requirement of a transcription-coupling factor for TC-NER (Cockayne syndrome B protein [CSB], mutated in Cockayne syndrome complementation group B, a human condition associated with developmental defects and neurodegeneration), whereas GG-NER (but not TC-NER) requires the XPC factor, while the XPA protein is required for both pathways. What is more difficult to control is the nature of damage inflicted during genetic studies of ICL repair that rely on treatment with exogenous agents. These inevitably induce an array of lesions including ICLs but also monoadducts, intrastrand crosslinks, and DNA-protein crosslinks. Mulderrig and Garaycoechea dealt with this issue by studying the effects of FA and NER pathway loss in mice not exposed to damaging agents so that any observed spontaneous phenotypic consequences reflect endogenous damage and normal physiology.

Mulderrig and Garaycoechea created mice lacking ERCC1 (and, therefore, its obligate partner XPF) or XPA (eliminating all NER) or inactivated the FA pathway through disruption of *Fanca*. While *Ercc1*^*-/-*^ mice exhibited the expected hallmarks of liver damage and dysfunction, along with impaired renal function, these organs developed normally in both the *Xpa*^*-/-*^ and *Fanca*^*-/-*^ mice. *Ercc1*^*-/-*^ mice also showed the expected hematopoietic stem cell defects, which were more pronounced than in *Fanca*^*-/-*^ mice and absent in (young) *Xpa*^*-/-*^ mice. To test whether the more severe phenotype of *Ercc1*^*-/-*^ mice could be explained by dual inactivation of NER and FA pathways, mice doubly disrupted for *Xpa* and *Fanca* were created. While postnatal growth of *Ercc1*^*-/-*^ mice is drastically affected, *Xpa*^*-/-*^ *Fanca*^*-/-*^ mice were indistinguishable from *Fanca*^*-/-*^ single disruptants, exhibiting none of the liver or kidney abnormalities observed in *Ercc1*^*-/-*^ mice. Turning their attention to the hematopoietic system, disruption of *Xpa* in the *Fanca*^*-/-*^ background did not exacerbate FANCA-loss–associated hematopoietic stem cells defects, but *Ercc1*^*-/-*^ animals exhibited a more severe defect than any *Xpa*^*-/-*^ *Fanca*^*-/-*^ animals. Together, these observations indicate that, in the face of endogenous DNA damage, the developmental and degenerative features of *Ercc1* disruption cannot be simply accounted for by dual loss of the NER and FA pathways.

They then exposed a human leukaemic cell line (HAP1) to a variety of exogenous ICL-producing agents. As expected, loss of XPA conferred sensitivity to the drugs cisplatin and mitomycin C. In contrast to XPA and CSB disruptants, cells defective in XPC (required for GG-NER) displayed no increased sensitivity to these drugs, implying an important role for TC-NER in helping cells survive crosslinks. Interestingly, *XPA*^*-/-*^ *FANCL*^*-/-*^ cells demonstrated epistatic sensitivity to mitomycin C relative to the cognate single disruptants. This could imply that FA and NER pathways cooperate in the repair of a subset of ICLs. For the two aldehydes implicated in generating endogenous ICLs, formaldehyde and acetaldehyde, the data suggested that these induce a distinct spectrum of lesions: NER (*XPA*^*-/-*^*)* and FA (*FANCL*^*-/-*^*)* defective cells were both sensitive to formaldehyde in a nonepistatic manner, whereas NER defective cells were not acetaldehyde sensitive, and NER loss did not enhance the sensitivity of acetaldehyde FA defective cells. Importantly, *XPF*^*-/-*^ cells were more sensitive than the *FANCL*^*-/-*^ and *XPA*^*-/-*^ single or double mutants in response to acetaldehyde, mitomycin C, and cisplatin, underlining the existence of an additional role for XPF–ERCC1 in repairing the crosslinks these induce. By contrast, *FANCL*^*-/-*^ *XPA*^*-/-*^ doubly deficient cells were similarly sensitive to *XPF*^*-/-*^ when treated with formaldehyde, which could indicate that the lesions induced by formaldehyde are removed by the NER and FA pathways.

What of the more dramatic phenotype of the XPF–ERCC1 null animals compared to those inactivated for both FA and NER pathways? One interpretation of the mouse studies carried out by Mulderrig and Garaycoechea is that that XPF–ERCC1 is controlling cell survival though processes distinct from repair, such as regulation of gene expression [20]. However, a more likely culprit is a yet-to-be identified endogenously generated DNA lesion. Moreover, CSB, and a transcription-coupled repair pathway outside of NER might be involved, as mice jointly defective for CSB and XPA have a shortened life span and dramatic progeroid features compared to their cognate single disruptants [21, 22].

**Fig 1:**
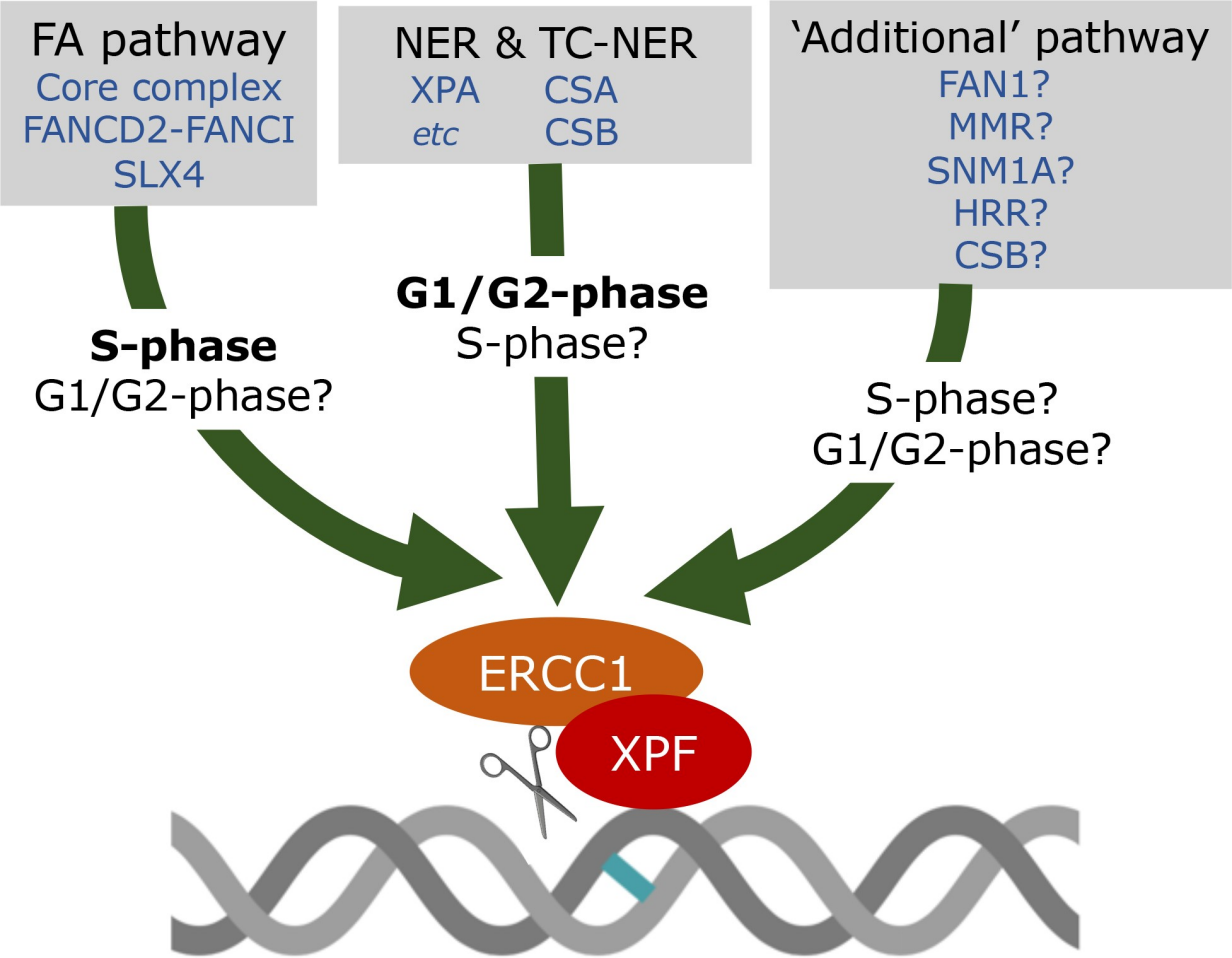
Multiple routes to the initiation of ICL repair require XPF–ERCC1. In mammalian cells, the FA pathway is the predominant activity orchestrating ICL repair during S-phase, although a role for NER is not excluded. A role for NER in the repair of ICLs in G1-phase of the mammalian cell cycle has been demonstrated, but it is unclear whether the FA pathway can contribute outside of S-phase. The work of Mulderring and Garaycoechea underlines the important role for TC-NER in response to ICLs. The extreme sensitivity of XPF-ERCC1 deficient cells, outside of its role in either NER and FA pathways, implies the existence of additional repair pathway(s) for counteracting ICLs. These could involve (in a nonexclusive manner), MMR, FAN1, SNM1A, CSB, and HRR events initiated by alternative endonucleases. CSA, Cockayne Syndrome A protein; CSB, Cockayne Syndrome B protein; FA, Fanconi anaemia; FAN1, Fanconi-associated nuclease 1; FANCD2-FANCI, Fanconi anaemia Group D2 and Fanconi anaemia Group I; G1-phase, Gap 1 phase; HRR, homologous recombination-based repair; ICL, interstrand crosslink; MMR, mismatch repair; NER, nucleotide excision repair; SLX4, structure-specific endonuclease subunit SLX4; SNM1A, sensitive to nitrogen mustard 1A; S-phase, synthesis phase; TC-NER, transcription-coupled NER.

The extreme ICL sensitivity of *XPF*^*-/-*^ human cell lines might be explained if the canonical ICL-repair pathways (FA and NER) fail, and ‘last resort’ repair events are initiated (Fig 1). These might involve alternative initiating endonucleases (MUS81 or SLX1, for example [23]) leading to homologous recombination steps requiring XPF–ERCC1 for their successful completion. Moreover, the FAN1 (Fanconi-associated nuclease 1) nuclease, mismatch repair system, and the SNM1A (sensitive to nitrogen mustard 1A) exonuclease have all been implicated in ICL recognition and processing [24–26] and are candidates for mediating any XPF–ERCC1-dependent pathway, noting that the cell-cycle phase might be critical (the study of ICL repair outside the synthesis phase [S-phase] has been relatively neglected). Fortunately, the last decade has seen the development of elegant cell-free ICL-repair systems using *Xenopus* cell extracts [26, 27] and structural- and biochemical-reconstitution approaches that will help accelerate our mechanistic understanding of new pathways as they are revealed.
